# World Spread of Tropical Soda Apple (*Solanum viarum*) under Global Change: Historical Reconstruction, Niche Shift, and Potential Geographic Distribution

**DOI:** 10.3390/biology12091179

**Published:** 2023-08-29

**Authors:** Yuhan Qi, Xiaoqing Xian, Haoxiang Zhao, Ming Yang, Yu Zhang, Wentao Yu, Wanxue Liu

**Affiliations:** 1 State Key Laboratory for Biology of Plant Diseases and Insect Pests, Institute of Plant Protection, Chinese Academy of Agricultural Sciences, Beijing 100193, China; qyh_nwnu@163.com (Y.Q.); xianxiaoqing@caas.cn (X.X.); hx_zhao@bjfu.edu.cn (H.Z.); y1750165592@163.com (M.Y.); zhangyuu960606@163.com (Y.Z.); 2 Fujian Key Laboratory for Technology Research of Inspection and Quarantine, Technology Centre of Fuzhou Customs, Fuzhou 350001, China

**Keywords:** climate change, invasive alien plants, *Solanum viarum*, land use change, ecological niche, ecosystem

## Abstract

**Simple Summary:**

*Solanum viarum* has been a widely invasive species. We aimed to understand the prevailing historical dispersal, ecological niche dynamics, and distribution patterns. The invasion history of *S. viarum* consisted of three phases: lagging (before 1980), dispersal (1980–2010), and equilibrium (2010–present). Ecological niches remained conservative. The potential geographic distribution of *S. viarum* will reach a maximum in the 2050s, SSP5–8.5, and in the future, it will migrate to higher latitudes. Global factors continue to increase this threat. The relevant quarantine authorities should take prevention and control measures.

**Abstract:**

*Solanum viarum* has become extensively invasive owing to international trade, climate change, and land–use change. As it is classified as a quarantine weed by countries such as the U.S. and Mexico, it is critical to understand the prevailing historical dispersal, ecological niche dynamics, and distribution patterns. We reconstructed the historical invasion countries and analyzed the ecological niche shift of *S. viarum*. Using MaxEnt based on the conservativeness of ecological niches, we studied variations in the potential geographical distributions (PGDs) of *S. viarum* in ecosystems and variations in suitability probabilities along latitudinal gradients. The invasion history in six continents involved three phases: lag (before 1980), spread (1980–2010), and equilibrium (2010–present). The ecological niche remains conserved. The area of *S. viarum* PGDs had increased by 259 km^2^; the PGDs will expand to reach a maximum in the 2050s, SSP5–8.5. The PGDs of *S. viarum* will migrate to higher latitudes under the same future climate scenarios. The latitudes subject to high threats range from 20° to 30° in forest and cropland ecosystems, 15.5° to 27.5° (northern hemisphere) and 33.1° to 42.8° (southern hemisphere) in grassland ecosystems, and 20° to 35° in urban ecosystems. Global change has led to an increased threat of *S. viarum* at high latitudes. These findings provide a theoretical basis to monitor and control *S. viarum*.

## 1. Introduction

Plant invasions impact ecosystem functions, including accelerated competition for resources and reduced native plant diversity [1,2]. Global factors, such as international trade, climate change, and land–use change (LUC), can act individually or together to exacerbate the threat of plant invasion [3]. International trade in propagules, seeds, live plants, and flowers directly leads to the introduction of alien plants to new target regions, reshaping the biogeography [4]. However, international trade also plays an indirect role by altering the natural resources and social environments of exporting and importing countries [5]. Climate change may affect the ability of alien plants to survive and reproduce, enhancing adaptations to colonize otherwise unsuitable areas, such as high latitudes [6]. LUC, such as urban expansion, causes habitat fragmentation and the destruction of native ecosystems which affect the richness and frequency of alien plants [7]; it provides vacant ecological niches and creates feasible conditions for the invasion of alien plants [8]. Moreover, international trade, climate change, and LUC have resulted in land pollution and dramatic urbanization [9,10], which have a combined effect on plant invasion. Understanding the role of global changes in plant invasion is essential for ensuring sustainable development.

*Solanum viarum* Dunal (Solanaceae), commonly known as tropical soda apple, is a perennial seed propagated weed [11] native to South America (Brazil, Paraguay, Uruguay, and Argentina) and is now widely distributed (except Antarctica) [12]. *Solanum viarum* is spread via three main ways, namely as a popular medicinal plant by anthropic activity (international trade), as fruits by animals (e.g., birds and cattle), and naturally by vectors such as wind or water [13]. The species has been observed in pine forests, pastures, citrus plantations, and urban riparian habitats [14]. The species loss, health damage, and economic costs it causes in different ecosystems are of great concern, despite its medicinal value in India [15]. The whole plant of *S. viarum* is spiny; in forest and cropland ecosystems, it often occupies the space of neighboring plants, affecting the growth of native plants and reducing the yield of commercial crops [16]. *Solanum viarum* contains toxic alkaloid chemicals. The fruits, especially the immature ones, are more toxic and may cause respiratory distress, weakness, and even death when ingested by humans or livestock in grassland and urban ecosystems [17,18]. Moreover, injuries to humans and livestock from thorny plants often occur. As a result, the United States Department of Agriculture declared it a troublesome invasive weed in 15 states in 2012; Mexico added it to the list of quarantine pests in 2018 and Chile added it to the list of quarantine pests in category A1 in 2019 [19,20].

Reconstruction of the invasion history and identification of potential geographical distribution (PGDs) are key components of risk assessment. Invasion history reconstruction can identify the temporal phases of invasion and trends in different phases [21]. Species distribution models (SDMs) are used to predict the PGDs of invasive species prior to the occurrence of an invasion and have the advantage of requiring only occurrence records and environmental variables to model the maximum probability of species distribution [22]. They are based on the assumption of “niche conservatism”. However, ecological niches may shift during the process of global change and interactions between organisms; thus, the extent of the niche shift (whether it is stable) needs to be assessed. The two–dimensional centroid shift, overlap, unfilling, and expansion (COUE) framework is a common method to assess niche shifts. Guisan et al. [23] first proposed the COUE framework and stated that it can be used for invasion studies (including plants and pathogens). Pili et al. [24] used the COUE framework to compare the realized ecological niches of four alien species and assessed the extent of niche conservatism. The assessment of the ecological niche shift can be used to validate MaxEnt model results, which guarantees the accuracy of the predictions. Additionally, most recent studies have focused on PGDs and ecological niche shifts of alien species under trade, climate change, or LUC alone [25,26], with some studies emphasizing how LUC is associated with future trade and climate change affects alien species [27,28,29]. However, the intricacies of PGDs and ecological niche shifts of alien species under simultaneous variation in all three have been neglected.

*Solanum viarum* causes serious damage to natural ecosystems and has a negative impact on ecological balance. Studying ecological niche shifts and PGDs under global change provides guidance for early warning systems. Here, we (1) reconstructed the historical invasion of *S. viarum* in three temporal phases, (2) calculated ecological niche shifts to ensure the predictions of the MaxEnt model, (3) predicted the global PGDs of *S. viarum* in different ecosystems under climate change in the 2030s and 2050s, and (4) analyzed the variation in suitability probabilities along latitudinal gradients in different ecosystems. This study will provide an ecological and theoretical reference for assessing the invasion and spread of *S. viarum*.

## 2. Materials and Methods

### 2.1. Occurrence Records and Reconstruction of Invasion

We obtained 2037 global occurrence records of *S. viarum* from the open literature and databases (Table S1). Thereafter, we removed the occurrence data that did not contain detailed geographic information. ENMTools were used to screen the occurrence data such that only one distribution point was retained within each 5 km × 5 km raster [30]. Finally, we obtained 1074 occurrence records of *S. viarum* to model the PGDs using SDMs.

To reconstruct *S. viarum* invasion, based on the available documented countries from the Centre for International Agricultural and Biological Sciences (CABI), European and Mediterranean Plant Protection Organization (EPPO), Global Biodiversity Information Facility (GBIF), and Web of Science (WOS), we confirmed that 157 countries worldwide do not have relevant databases, documentation, or detailed geographical information on the occurrence of *S. viarum*. The earliest recorded time for field sampling and documentation was assumed to be the time of invasion in each country. We used four native countries of *S. viarum* publicly listed by CABI as the starting points for the pathway. The global spatiotemporal dynamics of *S. viarum* invasions were reconstructed, considering invaded countries prior to 1970 as a category and invaded countries from 1970 to 2023 as a category (with 10–year intervals). Additionally, we investigated the dynamics of the number of invaded countries based on a time series to analyze the invasion trends of *S. viarum* in different phases. The geographical observations were set to the World Geodetic System 1984 geographic coordinate system and imported into ArcGIS.

### 2.2. Climate Variables

The geospatial raster layers of the climate variables were available in ArcGIS (ESRI), and 19 unique covariates were included in the SDM. These covariates for 1970–2000 were obtained from the PaleoClim database with a resolution of 5 arc minutes [31]. Future covariates were derived from the WorldClim database [32]. Three shared socioeconomic paths, SSP1–2.6, SSP2–4.5, and SSP5–8.5, were used for the 2030s and 2050s. These indicate low, medium, and high CO_2_ concentrations in future scenarios based on the BCC–CSM2–MR, respectively. When two covariates had Pearson correlations, that is, |r| > 0.8, only the larger contributor was selected for modeling.

### 2.3. Land–Use Harmonization Data

Land–use harmonization data (LUH2) (https://luh.umd.edu/ (accessed on 20 June 2023)) is the latest complete set of land–use scenario data that links historical reconstructions of land use with future predictions to study variations in the suitability of *S. viarum* in different ecosystems under climate change. The dataset provided land use types, potential type shifts, and valuable agricultural reference information at a 1 km resolution per year, covering the period of 2015 to 2100 [33]. Historical reconstructions of the land–use dataset from 2015 were considered to match the near–current climate dataset [34] and used as the near–current LUC dataset. Future datasets cover scenarios SSP1–2.6, SSP2–4.5, and SSP5–8.5 and cover the 2040s and 2060s. The land use types (i.e., the ecosystems of *S. viarum*) were selected and classified as forest (consisting of primary and secondary forests), cropland, grassland (consisting of natural and managed grazing), and urban areas. We predicted the PGDs and non–PGDs of *S. viarum* for the near–current and future periods and quantified the proportion of PGDs to that ecosystem type in each grid cell, thereby obtaining information on variations in *S. viarum* PGDs in different ecosystems.

### 2.4. Model Settings and Evaluation

We predicted the PGDs of *S. viarum* under near–current climate and future scenarios based on occurrence records and environmental variables (including climate data and harmonized land–use data) using the MaxEnt model, which performs better than the species distribution model. As the most important parameters of the MaxEnt model, the calibration of FCs and RM can significantly improve prediction accuracy [35,36]. To simulate species responses to covariates, MaxEnt uses parameters with five characteristics: linear, L; quadratic, Q; product, P; threshold, T; and hinge, H. The range of the RM settings was increased from 0.5 to 4, and interval tests were performed using 0.5 intervals. Candidate models were built using the ENMeval package in R [37]. Finally, the model with the most significant delta values was selected.

The receiver operating characteristic (ROC) curve is based on a series of different dichotomies (cutoff values or decision thresholds), with the true positive rate (sensitivity) as the vertical coordinate and false positive rate (1–specificity) as the horizontal coordinate. The area under the curve (AUC), defined as the area under the ROC curve, is a performance indicator of accuracy [38]. The closer the AUC value is to 1.0, the higher the authenticity of the assay. The probability (*p*) of *S. viarum* occurrence was generated in an ASCII raster and ranged from zero to one in the model results. We classified the geographical distributions of *S. viarum* into two categories based on cutoff values: non–PGDs (0 < *p* ≤ 0.23) and PGDs (0.23 < *p* ≤ 1).

### 2.5. Ecological Niche Comparison Method

Based on the framework originally proposed by Broennimann et al. [39], the COUE framework was used to study the ecological niche shift of alien species in native and invaded countries. In the COUE framework, the ecological niche overlap index *D* proposed by Schoener was used to compare the ecological niches occupied by *S. viarum* in its native and invasive ranges [39]. Specifically, the ecological niche overlap index *D*, ranging from 0 to 1, indicates the degree of overlap between the ecological niches of alien species in native and invaded countries; large values indicate a high degree of overlap between ecological niches. Principal component analysis was performed using real occurrence records, and 8000 pseudo–absence points were randomly generated within the native and invasive ranges of *S. viarum* (points with latitudes greater than 70° were removed). The native and invasive ranges included all climatic zones in which *S. viarum* survives [40,41].

Equivalence and similarity tests were performed using the method proposed by Warren et al. [42]. The ecological niche equivalence test was used to determine whether the ecological niches of the native and invaded countries were equivalent, that is, random points were reassigned and the ecological niche overlap index *D* was calculated in the native and invaded countries; the iterations were repeated 100 times to ensure high confidence in rejecting the original hypothesis. If the achieved niche was within the 95% confidence range of the simulated value, the original hypothesis could not be rejected (ecological niche equivalence). The ecological niche similarity test was used to verify whether the environmental niche occupied in one range was more similar to the environmental niche occupied in a range other than that expected. If the niche achieved is greater than 95% of the simulated value after 100 iterations, the environmental conditions of the native and invaded countries are more similar than expected.

We evaluated three indicators of ecological niche shifts: stability, expansion, and unfilling [23,39]. Stability is the ability of a species to adapt to environmental change, expansion is the extension of the range of a species’ ecological niche in a new habitat, and unfilling is a vacant ecological niche in a new habitat that is not occupied by a species. The ecological niche comparison method was performed in R using the ecospat package [43,44].

## 3. Results

### 3.1. Reconstruction of Invasion

**Figure 1:**
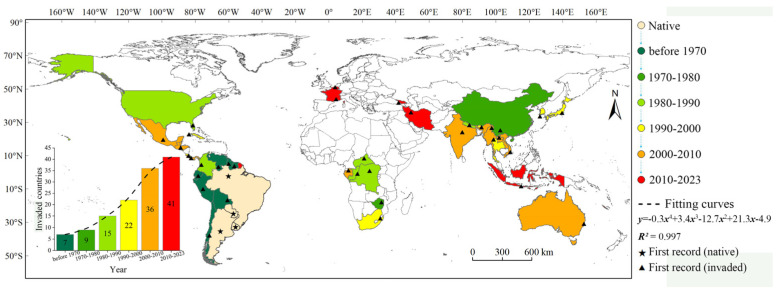
Reconstruction of *Solanum viarum* invasion. Six intervals are shown from green to red until 2023 (except for the native countries). The bottom left shows a composite graph of the quantities and trend of invaded countries.

The invasion history of *S. viarum* was grouped into three phases: lag (before 1980), spread (1980–2010), and equilibrium (2010–present), based on the characteristics of the temporal curve of the number of occurrence countries (Figure 1). The lag phase was a period of slow growth in the number of countries invaded by *S. viarum*, that is, the establishment phase. Prior to the 1970s, *S. viarum* was recorded as invasive in seven countries, and from 1970 to 1980, only two more countries were added. The spread phase was a period of rapidly increasing spread, during which the number of invading countries increased significantly. From 1980 to 1990, *S. viarum* invaded six additional countries, and from 1990 to 2000 *S. viarum* invaded seven additional countries. From 2000 to 2010, its spread peaked with *S. viarum* invading 14 countries. By 2010, *S. viarum* had spread to South America, southern North America, central Africa, Asia, and eastern Australia. The subsequent invasion by *S. viarum* reached a relative equilibrium. Since 2010, *S. viarum* has invaded five additional countries, for a total of forty–one countries, and is widely distributed across Asia, Europe, North America, South America, southern Africa, and Oceania. To date, 156 countries have not been affected by *S. viarum*.

### 3.2. Global Variation in the Ecological Niche of Solanum viarum

**Figure 2:**
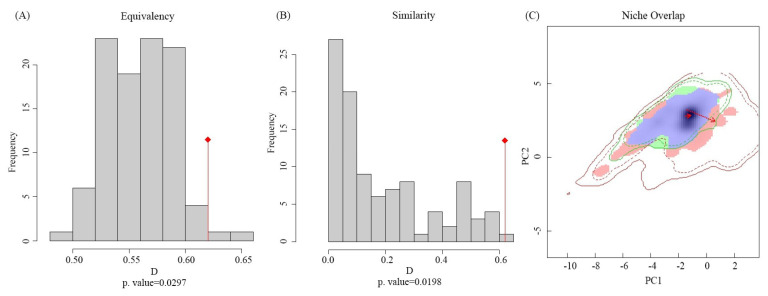
Global variation in the ecological niche of *Solanum viarum*. (**A**) *p* value of equivalency. (**B**) *p* value of Similarity. (**C**) Niche overlap: red and green blocks indicate expansion and unfilling areas, respectively, purple blocks indicate overlapping niches, and red arrow indicates variations in the niche centers of native and invasive areas.

The variation in the ecological niche of *S. viarum* was verified by comparing the differences in the ecological space between native and invaded countries (Figure 2). The results showed an ecological niche overlap index *D* of 0.62, indicating a high degree of ecological niche overlap between native and invaded countries. The equivalence (*p* = 0.0297) and similarity (*p* = 0.0198) tests indicated that *S. viarum* occupied a similar, but not identical, ecological niche in native and invaded countries. The values for the stability, expansion, and unfilling of the ecological niche were 0.951, 0.049, and 0.037, respectively. The ecological niche stability value was the highest and was greater than 0.95, indicating a conservative niche after invasion. Overall, the ecological niche of *S. viarum* expanded to a lesser extent and remained relatively stable.

### 3.3. Model Performance and Significant Variables

We chose the best model with significant delta values for the parameter optimization results of RM = 0.5 and FC = LQHPT (Figure S1). The AUC for the best model was 0.947 (Figure S2), indicating that the optimized MaxEnt model was excellent for predicting PGDs in *S. viarum*. Using the jackknife method (Figure S3), bio1, bio12, and bio7 were the three most significant variables. Based on an analysis of the variable contributions (Table S2), bio12, bio1, and bio3 were considered the three most significant variables. Therefore, the significant variables were three temperature factors and one precipitation factor (bio1, bio3, bio7, and bio12). Based on the response curves of the significant variables (Figure S4), the highest suitability probability value for *S. viarum* was 0.8 at an annual mean temperature of 20 °C. *Solanum viarum* had the highest suitability probability value of 0.85 when the value of isothermality was 53, annual temperature was 25 °C, and annual precipitation was approximately 1300 mm. Overall, the contribution of the LUC to the model was much smaller than that of the bioclimatic variables.

### 3.4. PGDs in Forest, Grassland, Cropland, and Urban Ecosystems

**Figure 3:**
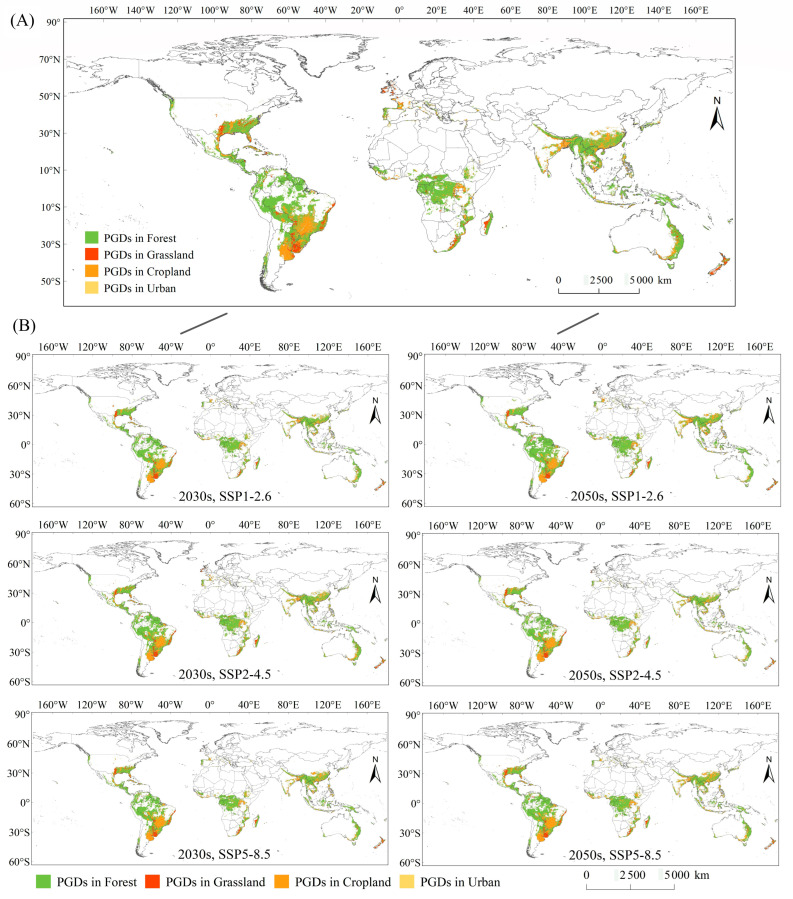
PGDs in forest, grassland, cropland, and urban ecosystems (extracted by land cover) under current climate scenario (**A**) and future scenarios (**B**).

The extracted PGDs of *S. viarum* in forests, grasslands, croplands, and urban ecosystems were distributed over southern and southeastern Asia, western Europe, southern and southeastern North America, Central America, northern and central South America, most of Central and Southeastern Africa, and eastern Oceania based on LUCs under near–current and future climate scenarios (Figure 3). Compared with those in the near–current climate, the predicted total global cropland and urban areas increased significantly under the future climate scenario, and the predicted total forest and grassland areas decreased to some extent (Table 1). However, the PGDs of *S. viarum* showed an expanding trend regardless of the four ecosystems in which they occurred, particularly in SSP5–8.5, in which the area of *S. viarum* PGDs reached a maximum in the 2030s and 2050s.

Under the current climate scenario, the PGDs of *S. viarum* in the forest were mainly in eastern and southern Asia, southeast Asia, eastern North America, Central America, northern and eastern South America, central and southeastern Africa, and eastern Australia, covering an area of 1467.42 km^2^. The geographical pattern of *S. viarum* PGDs in the forest did not change significantly under the future climate scenario, but the area of PGDs continued to increase and reached a maximum of 1748.15 km^2^ under the scenario SSP1–2.6 in the 2050s. Under the current climate scenario, the PGDs of *S. viarum* in grasslands were mainly found in southern North America, central and eastern South America, southern Africa, and New Zealand, covering an area of 151.83 km^2^. Under future climate scenarios, the PGDs of *S. viarum* in grasslands were mainly in southern North America, central and eastern South America, southern Africa, New Zealand in Oceania, and western Europe, with the area reaching a maximum of 159.98 km^2^ under the SSP2–4.5 scenario in the 2030s. Under the current climate scenario, the PGDs of *S. viarum* in croplands were mainly in southern Asia, south–central North America, south–central South America, central Africa, southwestern Europe, and eastern Australia, covering an area of 417.49 km^2^. Under future climate scenarios, the PGDs of *S. viarum* in croplands were mainly in southern and southeastern Asia, south–central North America, south–central South America, central and southeastern Africa, southwestern Europe, and eastern Australia, with a maximum area of 576.79 km^2^ under the SSP5–8.5 scenario in the 2050s. Under the near–current climate scenario, the PGDs of *S. viarum* in urban areas were mainly in southeastern North America and eastern South America, covering an area of 15.25 km^2^. Under future climate scenarios, the PGDs of *S. viarum* in urban areas were mainly in eastern Asia, southeastern North America, and eastern South America, with a maximum area of 30.53 km^2^ under the SSP5–8.5 scenario in the 2050s. Owing to the relatively small area of urban ecosystems globally, *S. viarum* also had fewer PGDs in urban areas, but not at a lower percentage.

### 3.5. Variations of PGDs in Forest, Grassland, Cropland, and Urban Ecosystems

**Figure 4:**
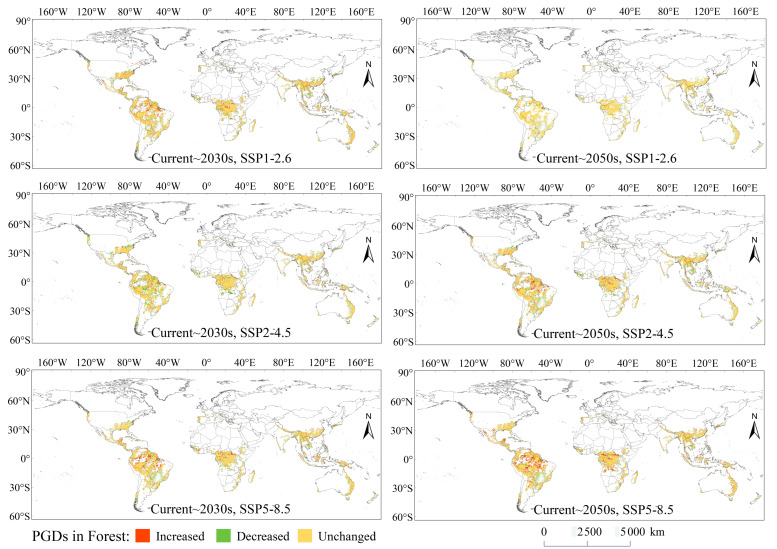
Variations of PGDs in forest under climate changes.

In the forest ecosystems (Figure 4), future increases in PGDs were mainly located in northern South America (Peru, Colombia, Venezuela, Brazil, Guyana, and Suriname) and central Africa (Congo and the Democratic Republic of Congo), with more significant increases in the 2050s. The largest area of increased PGDs was 456.53 km^2^ under the SSP5–8.5 scenario. Future decreases in PGDs were mainly located in eastern North America (USA), central South America (Brazil, Bolivia, Paraguay, and Argentina), central Africa (Central Africa, Gabon, Democratic Republic of Congo, and Tanzania), and southeastern Asia (China, India, Cambodia, Malaysia, and Indonesia), with more significant decreases in the 2050s. Under the SSP5–8.5 scenario, the decreases in PGDs reached the maximum area of 296.25 km^2^.

In the grassland ecosystems (Figure S5), future increases in PGDs were mainly located in southern North America (USA) and northern South America (Venezuela and Brazil), with more significant increases in the 2050s, and the largest area of increased PGDs was 51.06 km^2^ under the SSP5–8.5 scenario. Future decreases in PGDs were mainly located in eastern North America (USA), central South America (Bolivia, Paraguay, and Argentina), western Europe (Ireland, UK, and France), central Africa (Central Africa and DRC), and Australia, with more significant decreases in the 2050s. The largest decreases reached an area of 71.86 km^2^ under the SSP5–8.5 scenario.

In cropland ecosystems (Figure S6), future increases in PGDs were mainly located in eastern South America (Brazil and Argentina), central (Guinea, Côte d’Ivoire, Nigeria, the Democratic Republic of Congo, and Uganda), eastern Africa (Ethiopia, Tanzania, Mozambique, and South Africa), eastern (China) and southern Asia (India, Myanmar, Thailand, Philippines, and Indonesia), and Australia, with a more significant increase in the 2050s. The largest increase in PGDs reached 231.72 km^2^ under the SSP5–8.5 scenario. Future decreases in PGDs were mainly observed in North America (USA), Western Europe (France, Spain, and Portugal), Central Africa (Democratic Republic of Congo and Tanzania), Southern Asia (Thailand and Vietnam), and Australia, with more significant decreases in the 2050s, and the largest area being 137.85 km^2^ under the SSP2–4.5 scenario.

In urban ecosystems (Figure S7), the area of the PGDs did not decrease but continued to increase, with distributions in southeastern North America (USA), eastern South America (Brazil and Argentina), western Africa (Nigeria), eastern Asia (China), and Australia. The increase was more pronounced in the 2050s. Under SSP5–8.5, the area of increased PGDs reached its largest, at approximately 51.06 km^2^.

### 3.6. Suitability Probabilities along Latitudinal Gradients

**Figure 5:**
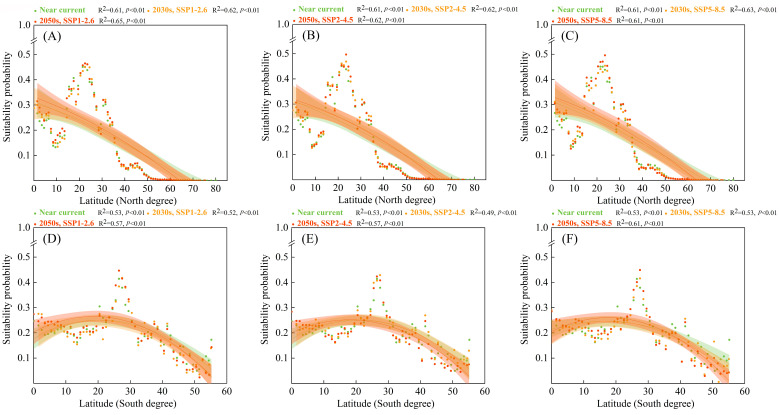
Suitability probabilities for *Solanum viarum* in the forest ecosystem under near–current and future climate scenarios (with 95% confidence bands via binomial fitting): (**A**,**D**) SSP1–2.6; (**B**,**E**) SSP2–4.5; (**C**,**F**) SSP5–8.5.

In the forest ecosystem (Figure 5), high suitability probabilities for *S. viarum* along latitudinal gradients were distributed within approximately 20–30° in the Northern and Southern Hemispheres. The high suitability probabilities for *S. viarum* shifted toward higher latitudes. In the Northern hemisphere, the highest suitability probabilities for *S. viarum* in the forest ecosystem were mainly located at 22.1°, whereas under the same future scenario, the highest future suitability probabilities for *S. viarum* were located at 23.5° under the SSP5–8.5 scenario in the 2050s, with an increase of 1.4°. In the Southern hemisphere, the highest suitability probabilities for *S. viarum* were mainly located at 26.5°, whereas under the same future scenario, the highest suitability probability for *S. viarum* was located at 27.9°.

**Figure 6:**
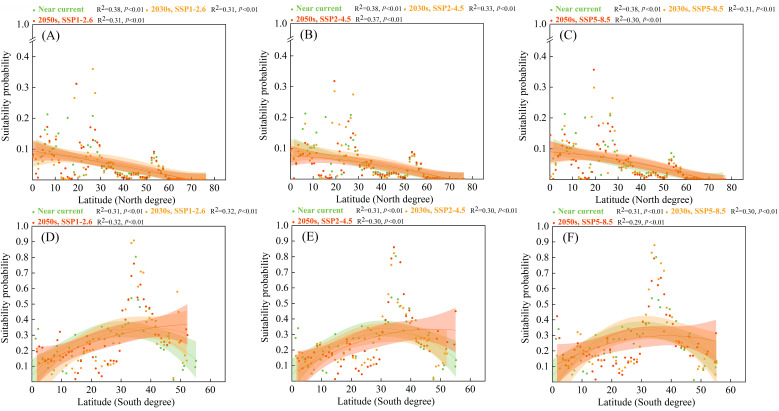
Suitability probabilities for *Solanum viarum* in the grassland ecosystem under near–current and future climate scenarios (with 95% confidence bands via binomial fitting): (**A**,**D**) SSP1–2.6; (**B**,**E**) SSP2–4.5; (**C**,**F**) SSP5–8.5.

In the grassland ecosystem (Figure 6), the distribution of high suitability probabilities for *S. viarum* in the Northern and Southern hemispheres differed significantly with latitude. However, the high suitability probabilities of *S. viarum* shifted toward higher latitudes. In the Northern hemisphere, the highest suitability probabilities for *S. viarum* in the grassland ecosystems were mainly located from 15.5° to 27.5°, and toward 30° in the future. In the Southern hemisphere, the highest suitability probabilities for *S. viarum* were located from 33.1° to 42.8° and toward 40° in the future.

**Figure 7:**
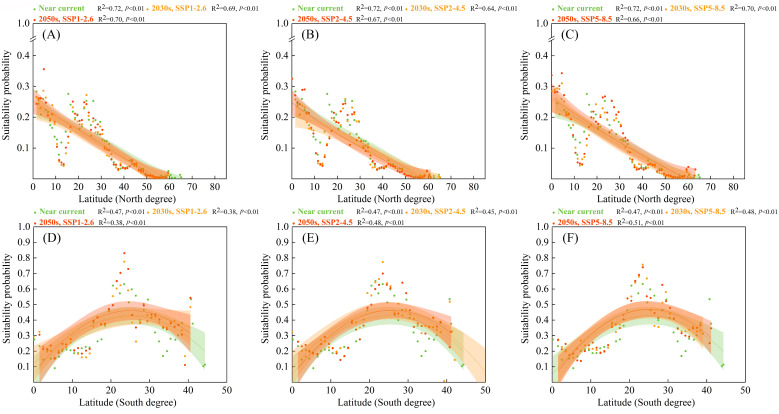
Suitability probabilities for *Solanum viarum* in the cropland ecosystem under near–current and future climate scenarios (with 95% confidence bands via binomial fitting): (**A**,**D**) SSP1–2.6; (**B**,**E**) SSP2–4.5; (**C**,**F**) SSP5–8.5.

In the cropland ecosystem (Figure 7), the high suitability probabilities for *S. viarum* along the latitudinal gradients were distributed within approximately 20–30° in the Northern and Southern hemispheres. The high suitability probabilities for *S. viarum* shifted toward higher latitudes. In the Northern hemisphere, the high suitability probabilities for *S. viarum* in the cropland ecosystem were mainly located at 4.5° and 23.5°, shifting toward 25° under the SSP5–8.5 scenario in the 2050s. In the Southern hemisphere, the high suitability probabilities for *S. viarum* in the cropland ecosystem were mainly located at 21.6° and 24.2°, shifting toward 25° under the SSP5–8.5 scenario in the 2050s.

**Figure 8:**
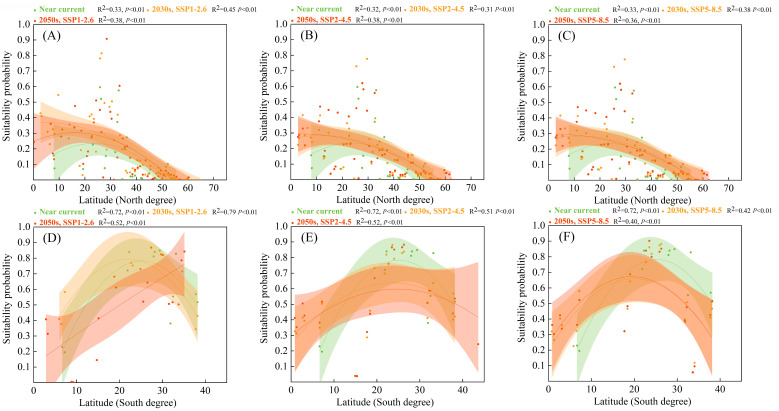
Suitability probabilities for *Solanum viarum* in urban ecosystem under near–current and future climate scenarios (with 95% confidence bands via binomial fitting): (**A**,**D**) SSP1–2.6; (**B**,**E**) SSP2–4.5; (**C**,**F**) SSP5–8.5.

In the urban ecosystem (Figure 8), high suitability probabilities for *S. viarum* along latitudinal gradients were distributed within approximately 20–35° in the Northern and Southern hemispheres. The high suitability probabilities for *S. viarum* were dense in urban ecosystems and reached higher latitudes. In the Northern hemisphere, the high suitability probability for *S. viarum* in urban ecosystems shifted from approximately 30° to greater than 30°. In the Southern Hemisphere, the high suitability probabilities for *S. viarum* shifted toward 25° and even 30°. 

## 4. Discussion

The tropical soda apple (*S. viarum*) is a troublesome invasive plant, and its global invasion has disturbed biodiversity and economics. However, this issue has received insufficient attention. Previous studies have focused primarily on its biological properties and hazards [11,15]. Few studies have examined potentially suitable areas, yet they have focused on localized areas [45]. Therefore, the present study is the first attempt to reconstruct the global invasion history of *S. viarum*, verify its ecological niche conservation, and predict PGDs using MaxEnt to provide theoretical guidance for early warning and prevention.

### 4.1. Reconstruction of Invasion History

The spread of invasive species is classified into three phases (lag, spread, and equilibrium), which can help determine the stage and process of invasion [21]. Our study showed that these three phases also apply to the global invasion patterns of *S. viarum*. *S. viarum* is mainly carried by wind or rivers and was mis—introduced as a potential fruit in South America and nearby (a few countries in Africa and Asia) in the lag phase (before 1980) [13]. Archaeological evidence suggests that human activity began to expand in the mid–to–late Holocene; alien plants were often transported as food by contemporary humans [46]. However, this hypothesis remains controversial [47]. The number of countries that *S. viarum* spread to in this phase was small, and no large–scale spread had yet occurred. There was an increase in the spread of *S. viarum* during the spread phase (1980–2010), with several continents invaded (except for Europe and Antarctica). Global plant trade networks emerged in the 18th and 19th centuries when alien plants began to appear frequently in non–native wild areas, particularly in the 19th century [48]. Increased trade in species, the complexity of trade networks, improved long–distance connectivity, and new trade patterns contributed to *S. viarum* invasion [49]. As a major source of pharmaceutical compounds (especially solasodine), *S. viarum* established colonies along trade routes and rapidly expanded during this phase. *Solanum viarum* successfully spread to all continents (except Antarctica) in the equilibrium phase (2010–present). Although the number of countries affected by *S. viarum* continues to increase, its spread rate has decreased significantly. Because *S. viarum* occupies an invasive ecological niche, when the ecological niche reaches saturation, a relative equilibrium state is formed [50]. However, symbiotic and antagonistic relationships may have arisen between *S. viarum* and native species, that is, potential competition is reduced or expansion is inhibited [51]. Biological control and management measures may be implemented to limit and mitigate the spread of *S. viarum* [52]. The equilibrium phase of *S. viarum* invasion does not imply that it no longer poses a threat to native organisms and ecosystems. Therefore, early warning and prevention of *S. viarum* invasion are essential to protect the integrity of ecosystems.

### 4.2. Ecological Niches and PGDs in Different Ecosystem

Many invasive plants expand their range to adapt to global change, and the extent to which their ecological niche changes following invasion has become a focus of biogeographic research [53]. Our study concluded that *S. viarum* has rapidly expanded its invasive range globally but largely occupies the same realized ecological niches as its native ranges, indicating ecological niche conservatism. This is inextricably linked to the biological characteristics of *S. viarum* [45]. Because of its limited drought tolerance, *S. viarum* thrives under hot and humid conditions [54]. A lack of precipitation limits photosynthesis in *S. viarum* leaves, and annual precipitation determines whether it can grow normally during the dry season [18]. The range of annual precipitation to which *S. viarum* is adapted in native countries (e.g., Brazil) is 800–1800 mm, with little variation in invaded countries. This suggests that it occupies a similar ecological space after invasion and has evolved adaptively to the environment [55]. A key insight from these results is that ecological niche variations cannot be ignored when predicting the future spatial distribution of species. SDMs are commonly used to predict expansion or invasion based on the premise that species maintain their original ecological niche when colonizing new habitats [56]. The conservative ecological niche of *S. viarum* demonstrated the excellent predictive ability of the MaxEnt model and provided assurance for the prediction of PGDs in different ecosystems.

Global changes affect the PGDs of *S. viarum* in different ecosystems. Studies have shown that climate change is already destroying parts of forests, and this species continues to migrate to higher latitudes (even polar regions) [57,58]. This is consistent with the results of this study. This trend is expected to become more pronounced under future climate change [57,59,60]. International trade, climate change, and LUC jointly influenced the PGDs of *S. viarum*; however, the first two factors were more significant. Although international trade and climate change play a dominant role in the distribution of *S. viarum* on a large spatial scale (i.e., global), LUC exerts a greater influence on a small spatial scale [61]. In the future, urban land is expected to continue to increase because of urbanization and population booms. The cropland area will also expand because an increasing number of people are in urgent need of food [62,63]. Anthropogenic activity promotes *S. viarum* invasion. This is consistent with our prediction that the areas of PGDs in croplands and urban ecosystems will continue to increase. In the future, PGDs will continue to expand under global change, and the risk assessment of *S. viarum* will contribute to an in–depth understanding of its spread dynamics.

### 4.3. Early Warning and Prevention Efforts

Invasive plants can spread through both natural and anthropogenic means [13]. Human activities, such as international trade and travel, help them cross geographical barriers and be introduced to distant countries where they do not occur. Globalization may exacerbate this problem [64]. A previous study suggested that unintentional human carriage is responsible for the spread of *S. viarum* in Africa [45]. Therefore, countries with suitable conditions for *S. viarum* that have not yet been invaded, particularly the Philippines, New Zealand, the United Kingdom, and Ireland, which are major agricultural and livestock countries [65], should focus on the global trade of plant and livestock vectors that can transport *S. viarum* seeds, seedlings, and fruits. Countries at risk of invasion should adopt appropriate quarantine measures to prevent the introduction of weeds. For example, in New Zealand, centralized livestock management is the main method used to control this weed [66]. Countries already invaded by *S. viarum* should intensify on–farm control using hexazinone and imazapyr herbicides [67] or release the natural enemy *Gratiana boliviana* [14]. All countries need to establish more effective quarantine processes at international borders, which are essential for interrupting the international transmission routes of *S. viarum*. Future global changes will increase the threat to countries, such as the United States, Nigeria, China, and Australia. Because there is usually a time lag between large–scale outbreaks and observations [68], these countries should establish a sound system for early monitoring and timely control.

## 5. Conclusions

Under global change, *S. viarum* has become widespread across six continents (except Antarctica), with 156 countries remaining uninvaded. The invasion history of *S. viarum* went through a lag phase (before 1980), spread phase (1980–2010), and equilibrium phase (2010–present). Moreover, the ecological niche of *S. viarum* remains conserved. The main ecosystems in which *S. viarum* is suitable are forests, grasslands, croplands, and urban areas; croplands and urban areas will become more suitable in the future. The PGDs in all four ecosystems continued to expand and were expected to reach a maximum in the 2050s under the SSP5–8.5 scenario. The latitudes subject to high threats range from 20° to 30° in forest and cropland ecosystems, 15.5° to 27.5° (northern hemisphere), 33.1° to 42.8° (southern hemisphere) in grassland ecosystems, and 20° to 35° in urban ecosystems. Global change has led to an increased threat by *S. viarum* at high latitudes. Analysis of the invasion history, ecological niche shifts, and future distribution dynamics provides a more reliable risk assessment for *S. viarum*.

## Figures and Tables

**Table 1:** Area of PGDs in forest, grassland, cropland, and urban ecosystems under current and future climate scenarios.

Area (×10^4^ km^2^)	Forest			Grassland			Cropland			Urban		
	PGDs	Total		PGDs	Total		PGDs	Total		PGDs	Total	
Near current	1467	6069	24%	152	1450	10%	417	2010	21%	15	51	30%
2030s, SSP1–2.6	1512	6103	25%	144	1363	11%	432	1996	22%	23	73	32%
2030s, SSP2–4.5	1602	6006	27%	160	1436	11%	489	2116	23%	23	71	32%
2030s, SSP5–8.5	1475	5920	25%	148	1403	11%	502	2183	23%	24	77	31%
2050s, SSP1–2.6	1748	6123	29%	135	1267	11%	505	2026	25%	25	83	30%
2050s, SSP2–4.5	1456	5992	24%	147	1385	11%	468	2186	21%	26	81	32%
2050s, SSP5–8.5	1557	5875	27%	147	1403	10%	577	2224	26%	31	93	33%

## Data Availability

The data presented in this study are available in this article.

## Supplementary Materials

The following supporting information can be downloaded at: https://www.mdpi.com/article/10.3390/biology12091179/s1, Table S1: Occurrence records of *Solanum viarum* details; Table S2: Environmental variable contributions related to the distribution of *Solanum viarum*; Figure S1: Parameter optimization results of Maxent on the occurrence records of *Solanum viarum*; Figure S2: The Maxent model performance results; Figure S3: Results of the jackknife test of variable importance; Figure S4: Response curves of important variables; Figure S5: Variations of PGDs in grassland under climate changes; Figure S6: Variations of PGDs in cropland under climate changes; Figure S7: Variations of PGDs in urban under climate changes.
