# Supplementary material for: World Spread of Tropical Soda Apple (Solanum viarum) under Global Change: Historical Reconstruction, Niche Shift, and Potential Geographic Distribution
Source: Biology (Basel). 2023 Aug 29;12(9):1179. doi: 10.3390/biology12091179 (PMC10525411; doi:10.3390/biology12091179)
Supplement: Supplementary file 1 [file biology-12-01179-s001.zip › biology-2551995-supplementary.pdf]

Supplementary material

Table S1. Occurrence records of *Solanum viarum* details.

Source	Number of occurrence records
GBIF (http://www. gbif.org/)	1685
CVH (http://www.cvh.ac.cn)	22
NSII (http://www. nsii. org. cn)	10
Web of Science (https://www.webofscience.com/)	320

Table S2. Environmental variable contributions related to the distribution of *Solanum viarum*.

Variables	Percent contribution (%)
bio12	50.1
bio1	24.3
bio3	11
bio14	6.3
bio7	3.5
bio19	3.5
bio2	0.8
LUC	0.4

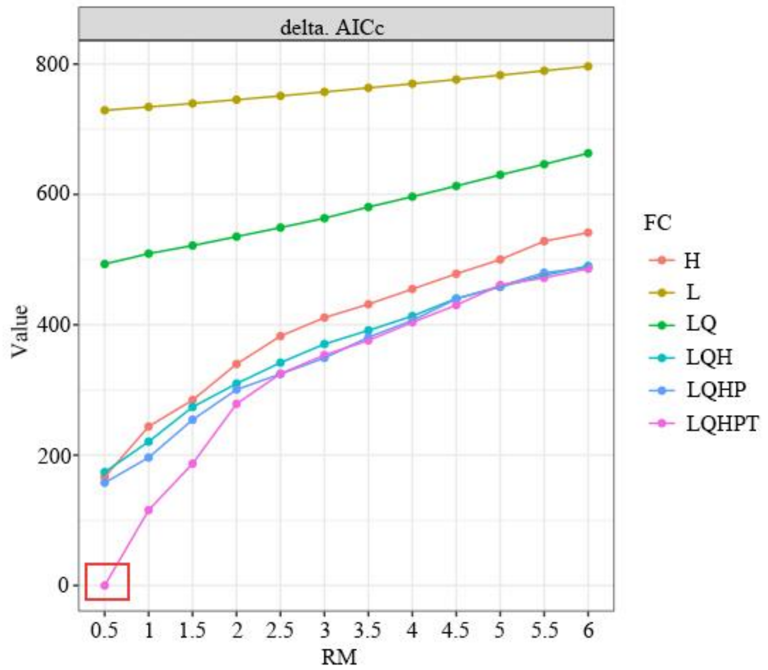

Figure S1. Parameter optimization results of Maxent on the occurrence records of *Solanum viarum*.

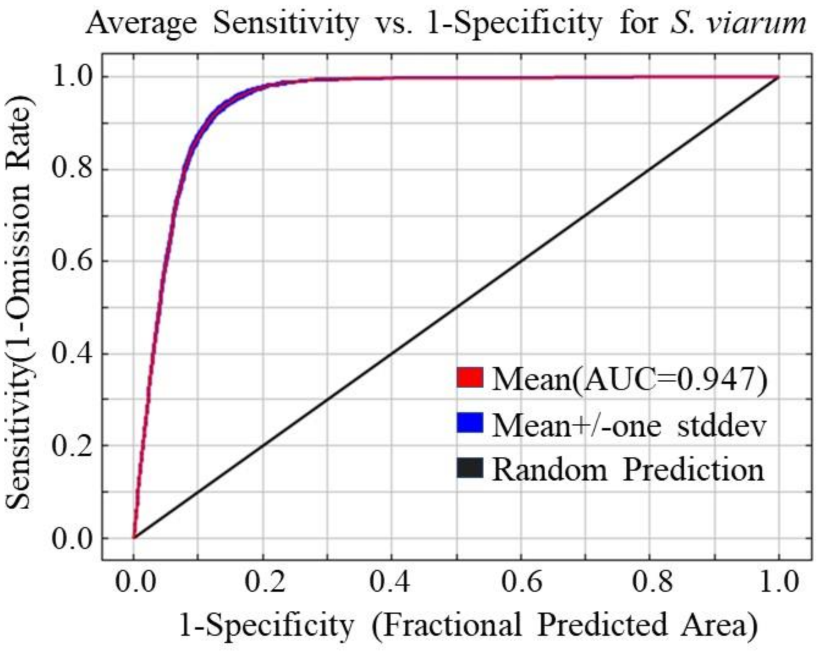

Figure S2. The Maxent model performance results.

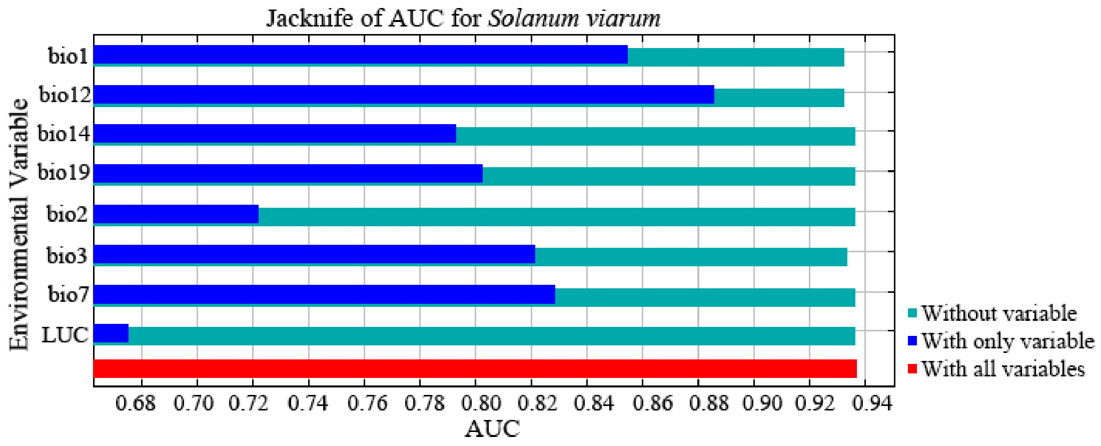

Figure S3. Results of the jackknife test of variable importance.

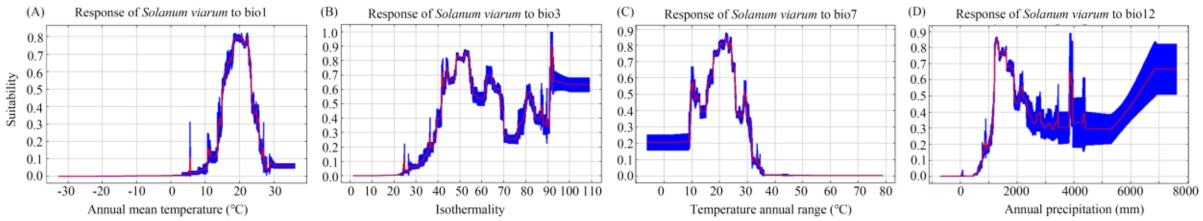

Figure S4. Response curves of important variables.

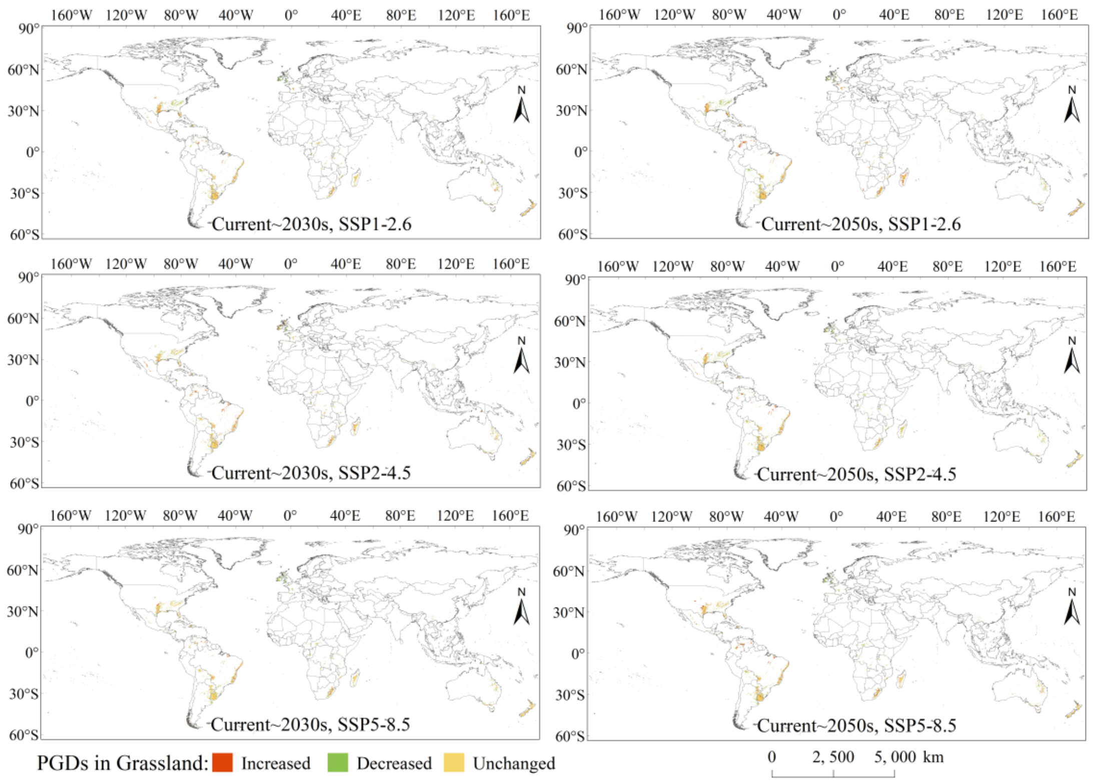

Figure S5. Variations of PGDs in grassland under climate changes.

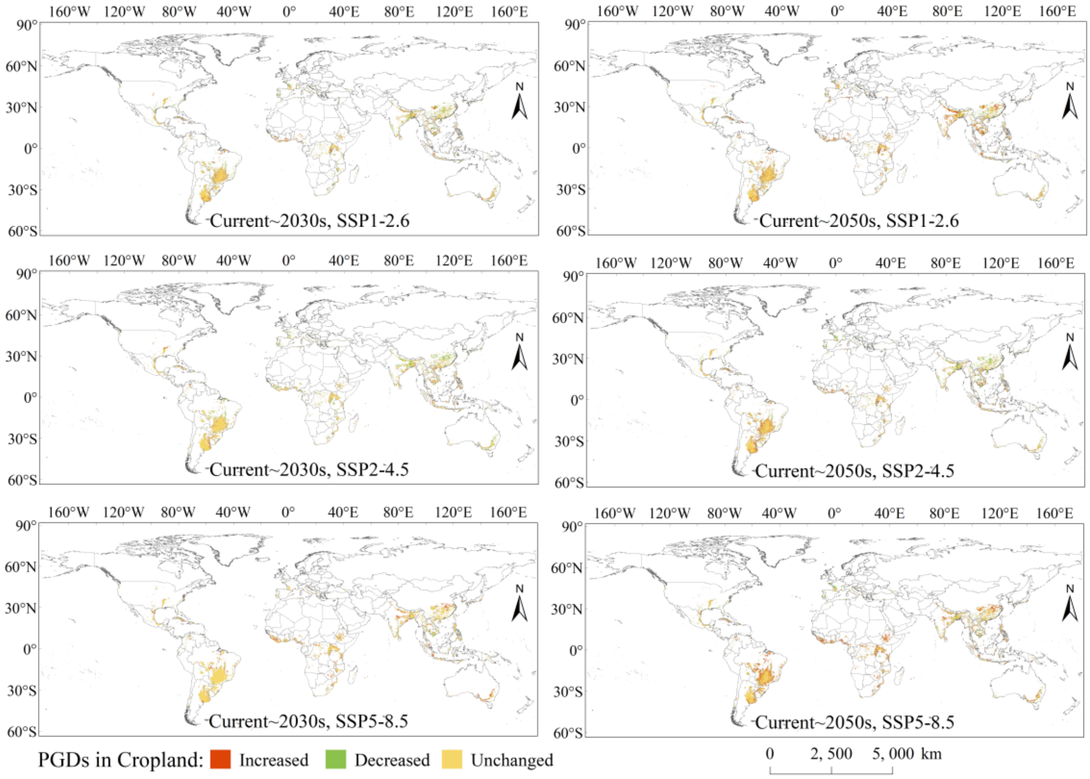

Figure S6. Variations of PGDs in cropland under climate changes.

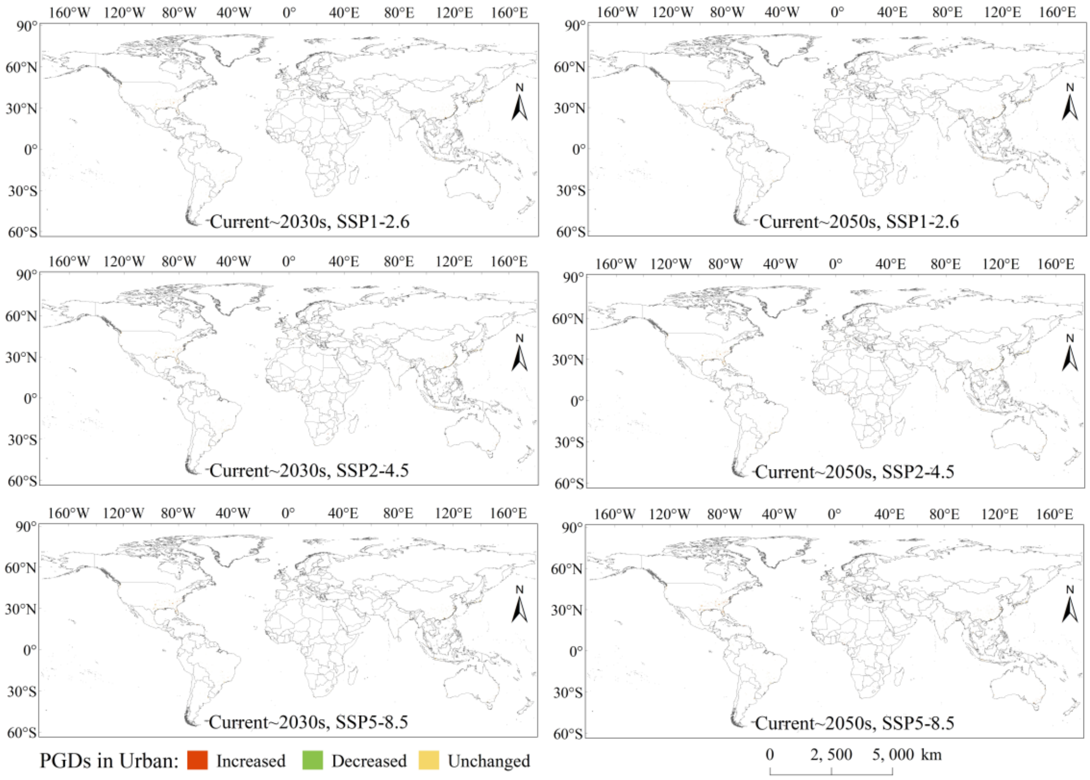

Figure S7. Variations of PGDs in urban under climate changes.
